# Live cell imaging reveals focal adhesions mechanoresponses in mammary epithelial cells under sustained equibiaxial stress

**DOI:** 10.1038/s41598-018-27948-3

**Published:** 2018-06-28

**Authors:** Lorena Sigaut, Catalina von Bilderling, Micaela Bianchi, Juan Eduardo Burdisso, Laura Gastaldi, Lía Isabel Pietrasanta

**Affiliations:** 1 Departamento de Física, Facultad de Ciencias Exactas y Naturales, Universidad de Buenos Aires and IFIBA, CONICET-UBA, Ciudad Universitaria, Buenos Aires, C1428EHA Argentina; 2 Centro de Microscopías Avanzadas (CMA), Facultad de Ciencias Exactas y Naturales, Universidad de Buenos Aires, Ciudad Universitaria, Buenos Aires, C1428EHA Argentina; 3 Consejo Nacional de Investigaciones Científicas y Técnicas (CONICET), Godoy Cruz 2290, Buenos Aires, C1425FQB Argentina

## Abstract

Mechanical stimuli play a key role in many cell functions such as proliferation, differentiation and migration. In the mammary gland, mechanical signals such as the distension of mammary epithelial cells due to udder filling are proposed to be directly involved during lactation and involution. However, the evolution of focal adhesions -specialized multiprotein complexes that mechanically connect cells with the extracellular matrix- during the mammary gland development, as well as the influence of the mechanical stimuli involved, remains unclear. Here we present the use of an equibiaxial stretching device for exerting a sustained normal strain to mammary epithelial cells while quantitatively assessing cell responses by fluorescence imaging techniques. Using this approach, we explored changes in focal adhesion dynamics in HC11 mammary cells in response to a mechanical sustained stress, which resembles the physiological stimuli. We studied the relationship between a global stress and focal adhesion assembly/disassembly, observing an enhanced persistency of focal adhesions under strain as well as an increase in their size. At a molecular level, we evaluated the mechanoresponses of vinculin and zyxin, two focal adhesion proteins postulated as mechanosensors, observing an increment in vinculin molecular tension and a slower zyxin dynamics while increasing the applied normal strain.

## Introduction

Under normal physiological conditions, cells are constantly subject to different external mechanical stimuli coming from neighboring cells or the surrounding extracellular matrix. Cellular response and adaptation to these mechanical stimuli are crucial in many cell functions as diverse as proliferation, differentiation and migration^1^. Moreover, several pathologies, such as cancer progression and metastasis, asthma or muscular dystrophies and cardiomyopathies^2^, can be associated with alterations or defects in how cells sense and transduce a mechanical stimulus into a biochemical signal, a process referred to as cellular mechanotransduction. Although many studies have been focused on this process, the precise mechanism by which external mechanical forces lead to eventual biochemical and molecular responses still remains unclear. Focal adhesions are specialized structures in which many of the biological responses to external forces are originated. These large and dynamic multiprotein complexes mechanically link the extracellular matrix to the cytoskeleton via integrin membrane receptors^3^. They exhibit mechanosensitive properties: their formation, development and disassembly are force-dependent and they have been postulated as signaling organelles in the cell mechanotransduction process^4,5^. Characterizing how these structures dynamically respond in the presence of a mechanical stimulus could lead to better understanding processes such as cell migration, motility and proliferation.

Cellular response to mechanical forces is multifaceted and diverse^6–8^, and may vary according to cell type and the way it is mechanically stimulated. Considering how external forces are applied and transmitted through the cell, as well as the magnitudes and distribution of the forces, is crucial in this kind of studies^9^. Moreover, a systematic study of cell mechanoresponses needs the mechanical stimulus to be controlled and highly reproducible. In this context, several mechanical stretching devices^10^ were developed and used for applying uniaxial or equibiaxial stress to cells in a sustained^11^ or cyclical manner^12,13^. Although many different kinds of mechanical stimuli can occur physiologically, the most widely studied is the cyclic uniaxial stretch. It has been shown that, in response to uniaxial cyclic stress, changes in the cytoskeleton and cell biochemistry depend on cell orientation relative to the direction of stretching, and cells tend to be reoriented perpendicular to the stretching direction^14^. However, tissues are as well commonly subjected to sustained stretch as for example in long-term blood pressure increase^15^, during prolonged muscle contraction^16^, or when a large volume of urine is retained in the bladder^17^. In particular, during the different stages of the mammary gland development, mammary epithelial cells are subjected to sustained mechanical stimuli such as the physical distention due to udder filling, or for example by the milk accumulation caused by the lack of suckling, which is known to trigger the expression and release of local factors that would initiate the mammary gland involution^18^. The evolution of focal adhesions during these stages, as well as how mechanical tension either from cell-cell or from cell-matrix interactions can affect its physiological influence is still unknown. In this context, mammary epithelial cells results an appealing model to study physiological and morphological changes in focal adhesions in response to an external, sustained equibiaxial mechanical stimulus, in terms to elucidate some cues on the mammary gland cell - matrix mechanical connection.

In this work, we present the use of a mechanical stretching device that allows sustained equibiaxial stretching of an elastic silicon membrane where cells are grown, while cell-responses are evaluated by several fluorescence microscopy and spectroscopy techniques. The controlled mechanical stretching produced by this device was characterized and found to be highly reproducible and efficiently transmitted to the cells. By imaging living cells expressing a fluorescently tagged adhesive protein, we were able to follow focal adhesion dynamics during the stretching experiments. Moreover, combining the use of the stretching device with advanced fluorescence imaging techniques we explored the effect of an external equibiaxial strain on two adhesion proteins postulated as mechanosensors: vinculin and zyxin. The studies on mechanoresponses to equibiaxial sustained stretch presented herein were all performed in the HC11 non-tumorigenic mouse mammary epithelial cell line^19^. These cells were previously found to exhibit responses to sustained stress, such as the ERK1/2 phosphorylation or by triggering involution associated cellular events^20^. Here we found an enhanced persistency of focal adhesions of cells under strain, as well as an increment on vinculin molecular tension and a slower zyxin dynamics while increasing the normal strain. Although mechanoresponses of vinculin and zyxin have been widely studied, the influence of a direct mechanical change of the substrate on these proteins at a molecular level has not been established before.

## Results

### A stretching device for live-cell fluorescence imaging: generation of a controlled and uniform equibiaxial strain

**Figure 1:**
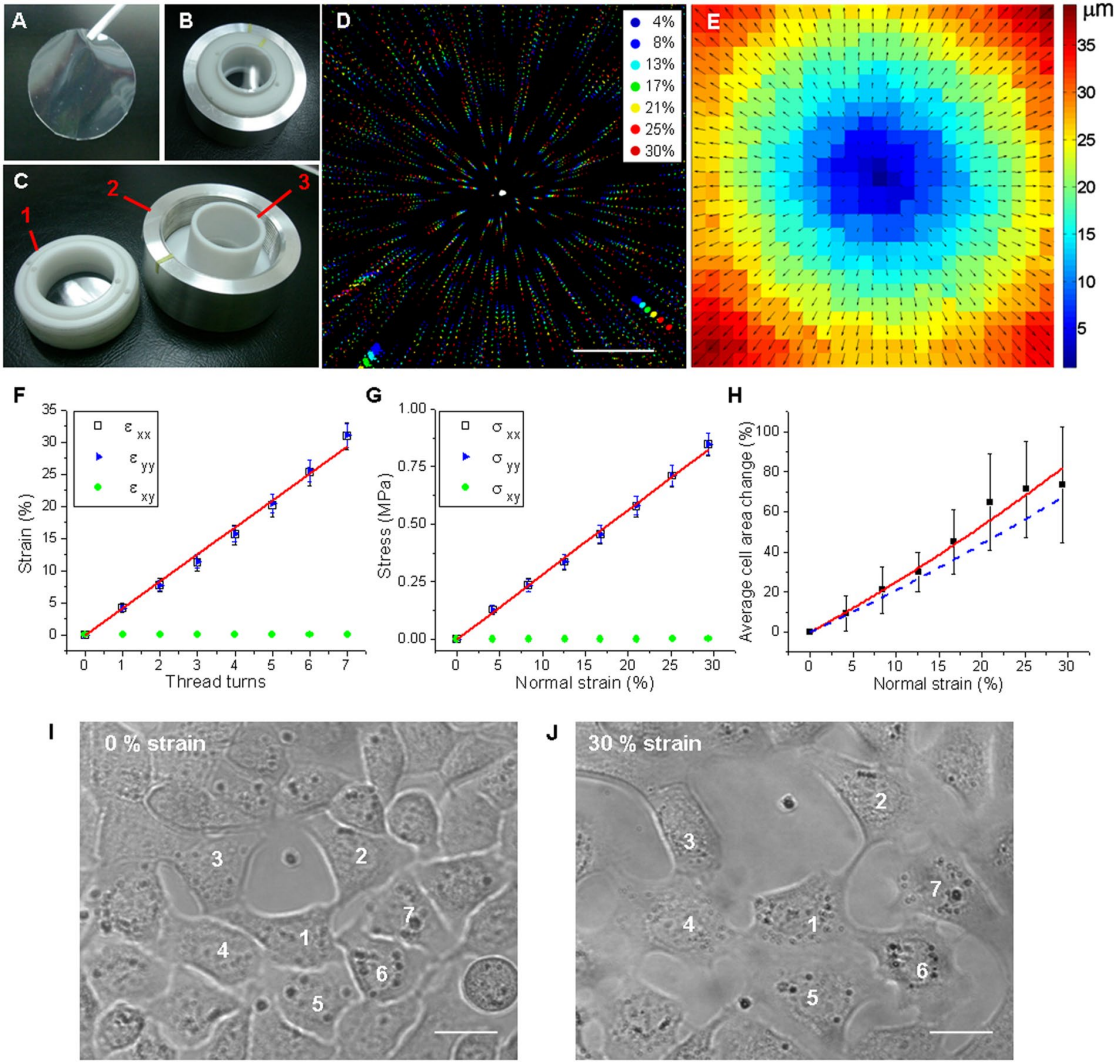
Equibiaxial stretching device. Flexible silicone membranes **(A)** are used as cell culture substrates for the stretching device **(B)**. Screwing the membrane holder piece **(C**-1) over the aluminum support **(C-**2) pushes the indenter ring **(C-**3) that stretches the silicone membrane. **(D)** Merged confocal image of fluorescent microspheres on a silicone membrane at increasing strain intensities, from 1 thread turn (4% strain) up to 7 thread turns (30% strain). Color code: normal strain percentage. Scale bar: 250 μm. Displacement vector map **(E)** obtained from PIV analysis of fluorescent images at 4% and 13% strain. Vector scale color code in μm. **(F)** Normal strain in *x* and *y* directions (open black squares and filled blue triangles, respectively), and shear strain (filled green circles) as a function of thread turns. A linear and homogeneous response was obtained in both normal directions and the shear strain was near zero. Red line is a linear fit of the normal strain components, slope (4.19 ± 0.08) % strain per thread turn. Data averaged from 3 independent experiments, s.d. as error bars. **(G)** Stress components as a function of strain: normal directions (open black squares and filled blue triangles) present the same behavior while shear stress (filled green circles) can be neglected. **(H)** Average cell area change as a function of strain (filled black squares). The red line corresponds to a quadratic fit while dashed-blue line the expected substrate area change estimated from the calibration. Average of N = 13 cells, s.d. as error bars. **(I-J)** Transmission images of HC11 cells at 0% **(I)** and 30% normal strain **(J)**, some of the cells are numbered as a reference. Scale bar: 20 μm.

A home made equibiaxial stretching device adapted from Quaglino *et al*. 2009^20^ for live fluorescence imaging was used to perform a mechanical stimulation to cells cultivated on flexible silicone membranes. The stretching produced by this device is based on an indenter ring that stretches a silicone membrane while being pushed by screwing the support (see Fig. 1A–C), and can be characterized in terms of thread turns.

The characterization and calibration of the equibiaxial strain was done by studying the displacement of fluorescent microspheres attached to the substrate as references. For this purpose, sequences of confocal images of increasing values of stretch from 0 (non-stretch) up to 7 thread turns were acquired. Using a particle image velocimetry (PIV) algorithm, the displacement maps were obtained and the strain and stress exerted on the silicone membrane by the stretching device at different stretching conditions were calculated. The merged image of a representative stretching sequence is shown in Fig. 1D, together with the displacement map estimated from confocal images at 1 and 3 thread turns (Fig. 1E). The average normal strain in *x*- and *y*- directions and shear strain were calculated and plotted as a function of thread turns (see Fig. 1F). As expected for an equibiaxial stretching device, the shear strain component was approximately zero and there was no significant difference between normal strain in *x* and *y* directions^21^. A linear and homogeneous response in both normal strain directions was obtained as a function of thread turns. The relationship between normal strain and the number of thread turns was obtained from the linear fit, which yields a slope of (4.19 ± 0.08) %. The shear stress can be neglected and the same linear behavior is observed in the normal directions of stress components (see Fig. 1G). This equibiaxial stretching device allows exerting up to 30% normal strain and a maximum normal stress of 0.85 MPa.

To determine whether mechanical strain was efficiently transmitted from the elastic substrate to the attached cells, the area of mammary epithelial living cells (HC11 cell line) was measured at increasing strain values and compared to the expected substrate change area. For this purpose a sequence of transmission microscopy images of living HC11 cells at increasing normal strain were analyzed. Figure 1I and J shows the transmission microscopy images of HC11 cells cultured on the membrane and stretched at 0% and 30% normal strain, respectively. At the highest strain, cells are still attached to the substrate and their area is increased. The average cell area for each strain condition as a function of the normal strain was plotted in Fig. 1H, together with the quadratic fit (red line) and the substrate area change, estimated from the calibration (dashed-blue line). The average cell area increased in response to the normal strain, following within the error, the substrate area increment. This result demonstrates that the mechanical stretching of the substrate produced by this device is effectively transmitted to the cells.

The use of both live fluorescence imaging and equibiaxial stretching of the cells cultured on silicone elastic membranes allowed us to deliver a controlled and reproducible mechanical stimulus giving rise to the following studies on focal adhesions mechanoresponses.

### Focal adhesions stabilize and grow under strain

**Figure 2:**
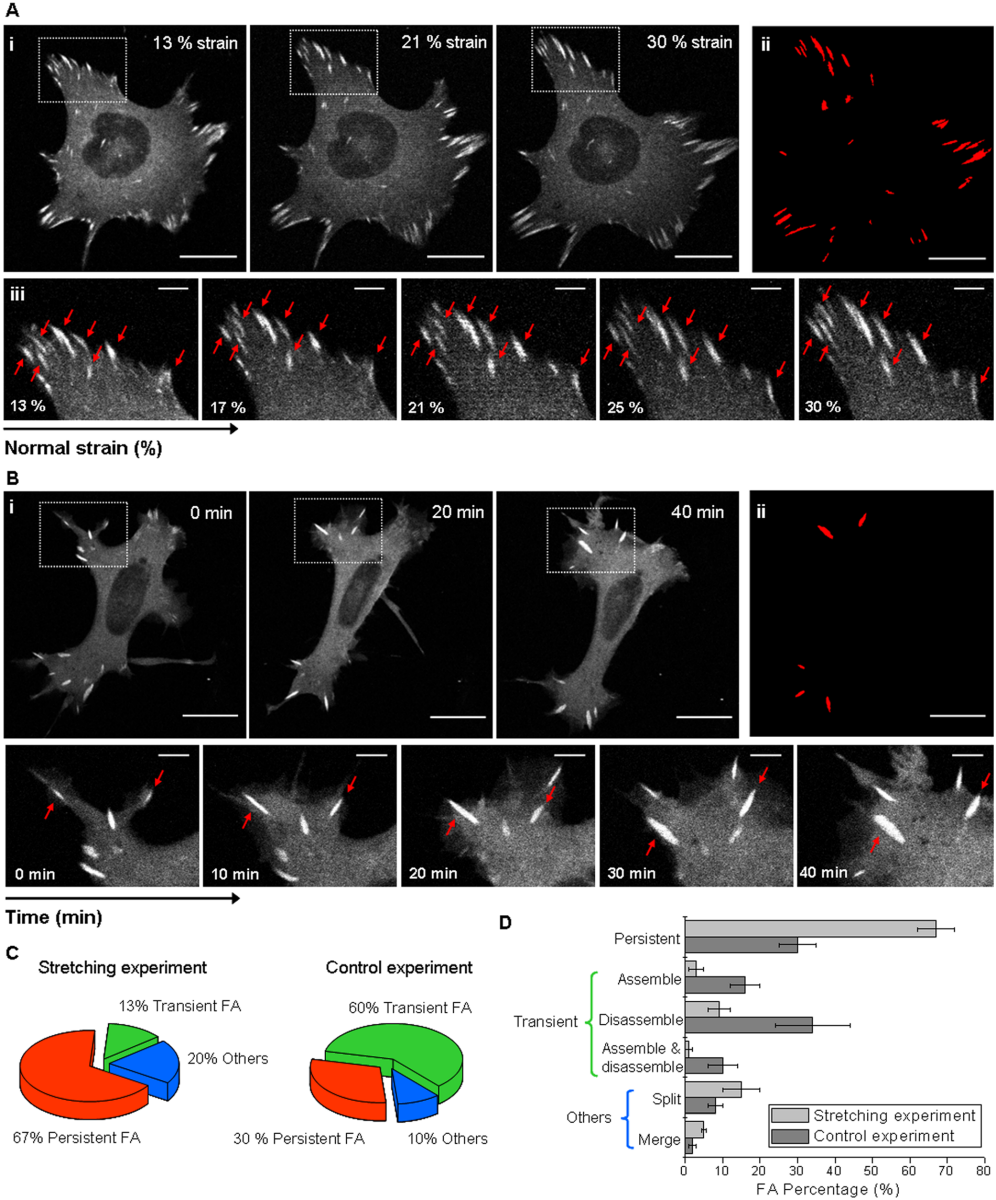
Focal adhesion dynamics in cells under strain. HC11 cells expressing a tagged fluorescent vinculin. **(A**i**)** Confocal image sequence of a cell at increasing normal strain from 13% up to 30%. **(B**i**)** A control sequence of images (time lapse 1 h) obtained as a function of time at strain 13%. **(A**iii) and **(B**iii) Sequential magnified view of the marked area in (Ai) and (Bi). Red arrows indicate persistent focal adhesions, defined as the ones that remain along the whole stretching or control experiment (approx. 1 h). Scale bar: 20 μm; zoomed images scale bar: 5 μm. **(A**ii**)** and **(B**ii) Maps of the persistent focal adhesions. **(C)** Proportion of focal adhesions according to theirs dynamics during stretching and control experiments (focal adhesions that are newly assembled and/or totally disassembled during the experiment are denominated as transient, and the one that emerged from a split event or disappeared from a merge event are classified as others). **(D)** Detailed percentages of persistent, newly assembled and/or totally disassembled, split and merged focal adhesion at increasingly stretched cells and in control cells. Data corresponding to N > 100 focal adhesions from 4 cells in stretching and control time lapse experiments were averaged; s.e.m. as error bars.

The effect of an external mechanical stimulus on the organization, dynamics and morphology of focal adhesions was evaluated on HC11 cells. For this purpose, confocal image sequences of HC11 cells transfected to express a fluorescent version of vinculin were acquired and analyzed while increasingly stretching the membrane by applying a normal strain from 13% up to 30%. As a control, confocal image sequences of cells were acquired at a constant strain of 13% during the time needed to perform the whole stretching experiment (approx. 1 hour). This control experiment allows discerning changes in focal adhesions related to stretching from changes due to the mere course of time during the experiment. Figure 2 shows a representative confocal image sequence of a cell that was increasingly stretched from 13% up to 30% normal strain (Fig. 2A) and a sequence of a control cell over time at a constant strain of 13% (Fig. 2B). Interestingly, focal adhesions have a tendency to persist when increasing strain is applied: there are less focal adhesions that are completely disassembled or newly formed. To better illustrate this tendency, Fig. 2(Aii) and (Bii) show the persistent focal adhesion maps for the stretching and control experiment, in which only focal adhesions that remained throughout the experiment, are represented. Here we defined persistent focal adhesions as the ones that remained along the whole stretching or control experiment (approx. 1 hour), and transient focal adhesions as those that were newly assembled and/or completely disassembled during the experiment. Focal adhesions that appeared as a result of a split event or disappeared because of a merge event with another focal adhesion were considered in the category others.

It is striking that, in cells subjected to increasing normal strains, 67% of the focal adhesions were persistent and only 13% were transient. By contrast, 30% of the focal adhesions of cells monitored over time (at a constant low strain) resulted persistent, while 60% were transient (see Fig. 2C). Regarding transient focal adhesions it is interesting to note that, of the entire population of focal adhesions from cells subjected to increasing strain, only 9% were completely disassembled, 3% were newly assembled and 1% were assembled and totally disassembled during the stretching experiment (see Fig. 2D). In control experiments, focal adhesions exhibited a considerable more transient behavior: 34% of the focal adhesions were completely disassembled, 16% were newly formed and 10% were formed and totally disassembled during the experiment. These findings suggest that equibiaxial cell stretching stabilizes focal adhesions preventing their disassembly, in agreement with counterpart experiments which evidenced a correlation between focal contact site disassembly and the retraction of the cell^22^, or a deficient cell spreading at low rigidity substrates^23^. Interestingly, in a previous work from the Waterman group^11^, focal adhesions from osteosarcoma cells subjected to a sustained uniaxial stretching were found to disassembly and the cell edge retracted when the cell’s long axis was oriented nearly perpendicular to the stretching direction. In the mentioned work, focal adhesions disassembly was observed to be prevented in cells whose long axis was nearly parallel to the stretching direction, whereas in our experiments, uniform equibiaxial stretching seems to prevent focal adhesions disassembly in all directions.

Additionally, focal adhesion morphometric changes generated by an increasing equibiaxial mechanical strain were followed in cells expressing a fluorescent version of vinculin. To this end, a semi-automatic identification and a morphometric characterization of persistent focal adhesions were performed in stretching or control sequences of images, and morphometric properties such as focal adhesion area, length and eccentricity were calculated at every image of a sequence.

**Figure 3:**
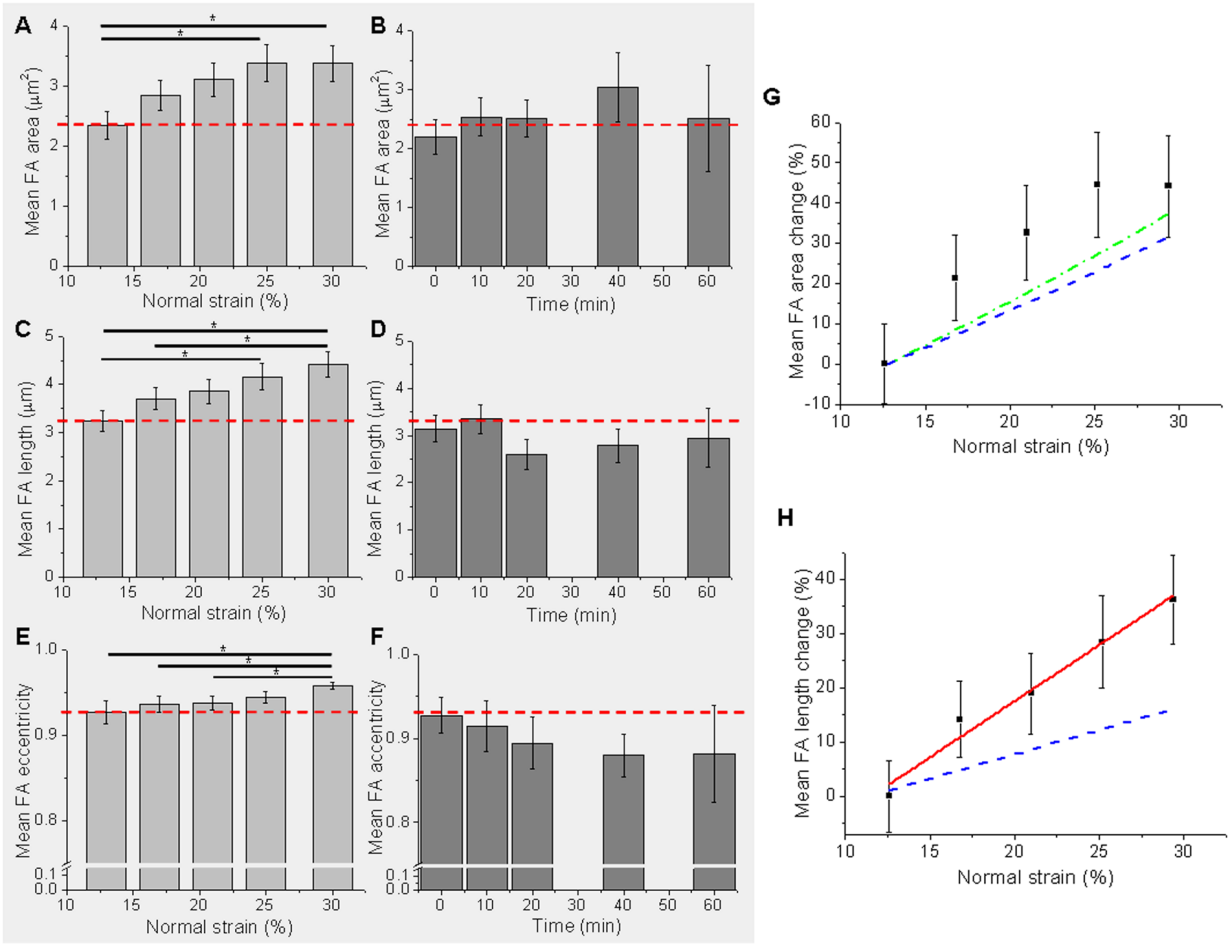
Quantifying changes in focal adhesion morphology. Mean focal adhesion area, length and eccentricity as a function of strain **(A)**, **(C)**, **(E)**, and as a function of time in control experiments at a constant strain of 13% **(B)**, **(D)**, **(F)**. As a reference, in horizontal dashed-red line, the value of the magnitude in the stretching experiment at the initial condition (13% normal strain). *Indicates p-value < 0.05, Mann-Whitney test between the conditions connected with a horizontal line. **(G)** Average focal adhesion area change as a function of strain. Expected substrate length change is shown in dashed-blue line, and expected cell area change in dotted-green line. **(H)** Average focal adhesion length change as a function of strain. Linear fit is presented in red line and expected substrate length change in dashed-blue line. N = 55 focal adhesions from 4 cells for stretching experiment, and N = 19 focal adhesions from 4 cells for control time lapse experiments; s.e.m. as error bars.

Figure 3 shows the average focal adhesion area, length and eccentricity as a function of normal strain from 13% up to 30% (Fig. 3A,C and E), and as a function of time in control experiments at a constant normal strain of 13% (Fig. 3B,D and F). At the initial condition, 13% strain, the average focal adhesion area was (2.34 ± 0.23) μm^2^. The strain increment resulted in a significant increase of focal adhesion area between the initial condition and normal strains of 25% and 30%. A significant raise of the mean focal adhesion length and eccentricity was also observed. On the contrary, in control experiments at constant 13% normal strain there were no significant changes, neither in the area, nor in the length or eccentricity of the focal adhesions during the time experiment, and their values were the same as the initial condition of the stretching experiment.

The relative change of the persistent focal adhesions mean area and length as a function of strain are shown in Fig. 3G and H, together with the expected area and length change of the substrate due to mechanical stretching of the membrane (dashed-blue line). We found that both focal adhesion area and length increased in response to strain, and that this increment is bigger than the mere increase due to the mechanical stretching of the substrate on which the cells are cultured. The growth of focal adhesions size induced by different mechanical strains was previously shown either in response to global stimuli like shear stress^24^, uniaxial^11,25^ or equibiaxial^25^ stretching (here in terms of fluorescently labeled adhesion proteins total intensity) or using substrates with modulated rigidity^23^; as well as in response to locally applied forces^26,27^. A key advantage in our experiments lies in the possibility of imaging the same cell at increasing equibiaxial stretching. Both live imaging and morphometric characterization would indicate that the mechanical tension applied by our equibiaxial stretching device is able to induce focal adhesions stabilization together with an increase in their size.

### Global mechanical stimuli modulate molecular mechanical tension across vinculin

The focal adhesion protein vinculin is crucial for the ability of cells to transmit forces and to generate cytoskeletal tension^28^. Vinculin molecule has a head and a tail domain that bind a number of focal adhesion and actin-regulating proteins actually connecting the cell to the substrate: while the tail domain binds to actin the head domain binds to proteins near the integrin receptors at the membrane. As vinculin is proposed to be responsible, among other functions, of transmitting external forces into the cells, it is interesting to address if mechanical tension among vinculin molecules can be modulated by a global substrate stretching.

**Figure 4:**
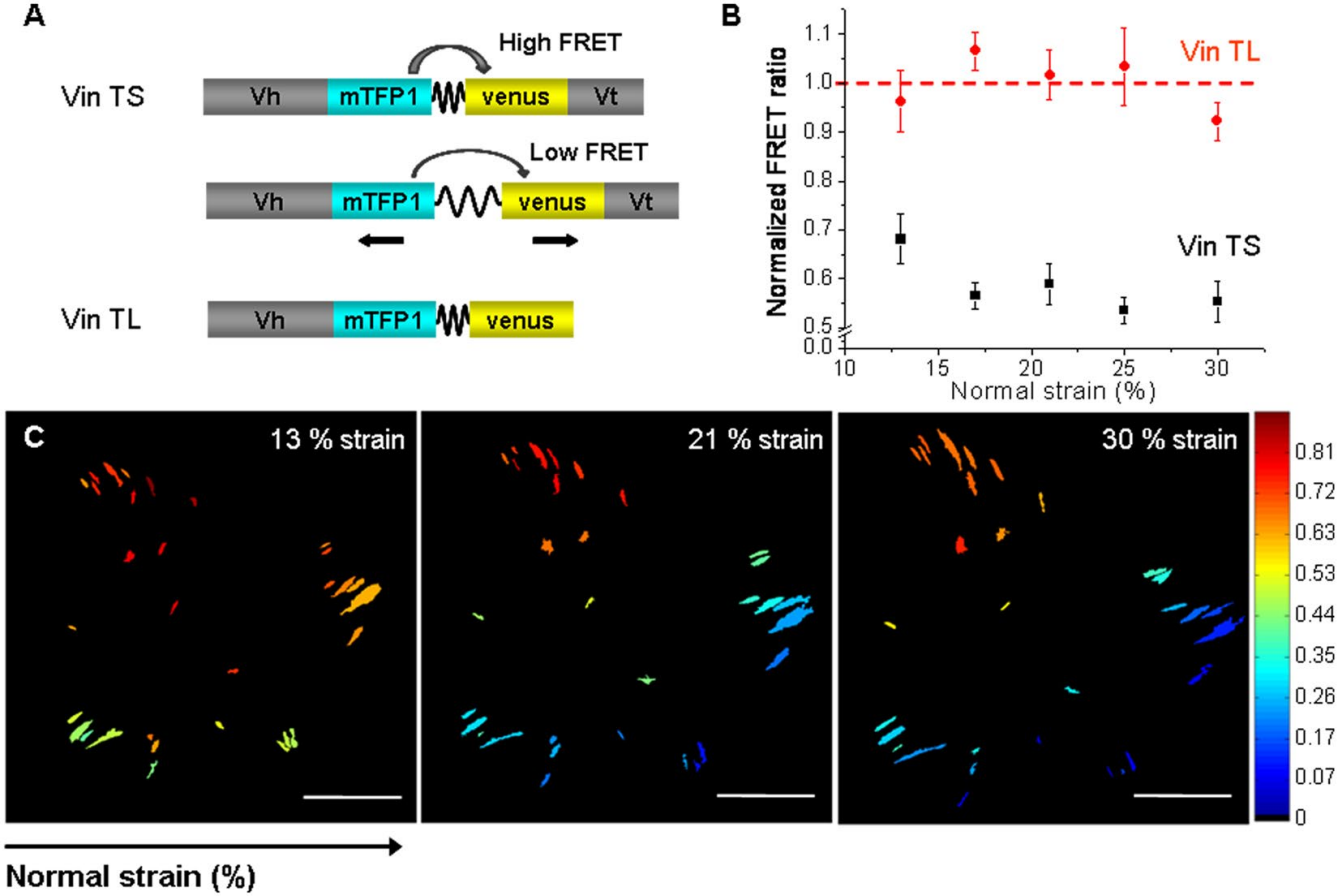
Mechanical tension changes across vinculin in response to mechanical stress. **(A)** The vinculin tension sensor (Vin TS) consists of a FRET pair fluorophores separated by a flagelliform linker inserted between the head (Vh) and tail (Vt) domains of vinculin. FRET between the donor fluorophore (mTFP1) and the acceptor fluorophore (Venus) is distance-dependent: when force across vinculin extends the elastic linker, FRET efficiency decreases. A construct without the tail domain, vinculin tail less (Vin TL), was employed as a positive FRET control (high FRET efficiency). **(B)** Average focal adhesion normalized FRET ratio as a function of normal strain, for Vin TS (black squares) and Vin TL (red circles). The normalized FRET ratio of N = 53–80 focal adhesions from 6 cells expressing Vin TS and N = 14–21 focal adhesions from 2 cells expressing Vin TL were averaged; s.e.m. as error bars. **(C)** Representative focal adhesion FRET ratio map sequence of a cell expressing Vin TS at increasing normal strain from 13% up to 30%. Scale bars: 20 μm. Color bar: normalized FRET ratio.

To measure changes in tension across this protein in focal adhesion of living cells a FRET biosensor developed by Dr. Grashoff and co-workers^29^, the vinculin tension sensor (Vin TS), was used. This tension sensor consists of a FRET pair of fluorophores separated by a flagelliform linker sequence inserted between the head and tail domains of vinculin molecule (see Fig. 4A). FRET between the donor fluorophore (mTFP1) and the acceptor fluorophore (Venus) is distance-dependent and therefore when force across vinculin molecule extends the elastic linker, FRET efficiency decreases. Tail less vinculin construct (Vin TL), without the tail domain, was employed as a positive FRET control. Since the tail domain is lacking, the linker is expected to remain unextended and the energy transfer to be maximum and constant. Being the stoichiometry of the donor and acceptor fixed, the ratio between the acceptor fluorescence and donor fluorescence signal was used to report changes in FRET efficiency. In this way, for each focal adhesion detected, a FRET ratio was calculated and then normalized considering the maximum and minimum FRET ratio signals estimated from control experiments employing Vin TL (maximum FRET ratio) and Vin mTFP (minimum FRET ratio, due to spectral crosstalk) (see materials and methods).

FRET experiments at increasing normal strains, were performed in HC11 cells expressing the vinculin tension sensor. If cells are well adhered and able to follow the substrate stretch, the simplest hypothesis could be that an equibiaxial strain would led to an increase of the molecular tension across vinculin protein within focal adhesions (*i*.*e*. an extension of the molecules as a result of an extension of the substrate). To test this hypothesis, for each detected focal adhesion, the normalized FRET ratio was calculated at different strain conditions. Figure 4C, shows a representative FRET ratio map sequence of a cell expressing Vin TS at increasing normal strain from 13% up to 30%. In these maps, the normalized FRET ratio at each focal adhesion is represented by a color according to the given color scale. Although there is a distribution of FRET values over the entire focal adhesions population of a cell at a given strain, for each individual focal adhesion there is a clear decrease in the FRET ratio value as the normal strain increases, *i*.*e*. a decrease of the energy transfer that corresponds to an increase of the molecular vinculin tension. As expected, molecular tension of vinculin seems to follow global substrate strain. To have an overall view, a mean focal adhesion normalized FRET ratio was calculated from tens of focal adhesions in different cells. Figure 4B shows the mean focal adhesion normalized FRET ratio as a function of normal strain, for Vin TS and Vin TL. As presumed, the mean normalized FRET ratio for the positive FRET control, Vin TL, remained approximately constant, regardless the normal strain applied. In addition, for all strain conditions the values of the mean normalized FRET ratio for the vinculin sensor (Vin TS) were significantly lower than the ones for the positive FRET control, implying that the flexible linker is not totally relaxed in the sensor. This difference indicates, as expected, that vinculin is in a tensioned state in focal adhesions of living HC11 cells. Moreover, this tensioned state tends to increase while normal strain increases, as it is suggested by the fact that the mean normalized FRET ratio for Vin TS has a tendency to decrease as normal strain increases. In particular, the dynamic range of the tension sensor, which was reported to be in the order of 10 pN^29^, seems to be covered within the smallest strains analyzed (less than 20% of normal strain). These findings suggest that vinculin in focal adhesions senses the global external stretching by direct increasing of its molecular tension state.

It is known that vinculin tension is altered by internal cell forces. According to Grashoff *et al*.^29^, highest tension across vinculin has been associated with adhesion assembly and enlargement in focal adhesions of migrating cells; and vinculin was found to be required for stabilizing adhesions under force during migration^30^. In previous works, employing the Vin TS construct, vinculin tension was shown to decrease with pharmacological inhibition or mechanical disruption of internal tension. For instance, the reduction of myosin induced traction forces, by pharmacological inhibition of the myosin activators ROCK (Y-27632) and/or MLCK (ML-7), resulted in a decrease of vinculin tension^29,31^. Moreover, the mechanical disruption of a single stress fiber by laser ablation, reduced tension across vinculin in focal adhesions, especially in focal adhesions oriented parallel to the targeted stress fiber^31^. Although changes on internal cellular forces has been proven to alter tension across vinculin proteins in focal adhesions, no studies have been previously reported on changes in vinculin molecular tension due to an external mechanical strain. The combination of the stretching device with live cell imaging of the vinculin biosensors, allowed a quantitative study of the changes in vinculin tension at focal adhesions in response to successively increasing normal strains. Our findings suggest that vinculin in focal adhesions can sense and respond at a molecular level to an external sustained mechanical stress, which in addition to previous studies about the influence of internal forces on vinculin tension, reinforce the fact that vinculin is one of the major force sensing and transmitting component of the cell-matrix interaction.

### Substrate stretching favors zyxin translocation to stress fibers

To get a deeper insight into adhesion proteins dynamics in response to externally equibiaxial strain applied to mammary cells, we studied the adhesion protein zyxin, known to be recruited at the final stages of the focal adhesion assembly^32^. Zyxin has been postulated as another mechanosensor protein, having a leading role in the cellular response to mechanical signals. Particularly, zyxin’s recruitment to stress fibers, also known as translocation, was proven to be mechano-dependent^33^. Previous findings have shown that the LIM protein zyxin responds to mechanical stress^33,34^ and regulates the remodeling and restoration of stress fibers through recruitment of multiple actin-regulatory binding partners^35^. Zyxin’s translocation to stress fibers can be induced by targeted application of mechanical stress via direct prodding with a microprobe^35^ or by reversibly stretching of single stress fibers by micromanipulation using atomic force microscopy^36^. A retrograde flux of zyxin along focal adhesions-anchored actin filaments is also found to be promoted by high substrate rigidity^37^, whether uniaxial cyclic stretch of cells was shown to produce a rapid and robust mobilization of zyxin from focal adhesions to actin filaments^33,38,39^.

**Figure 5:**
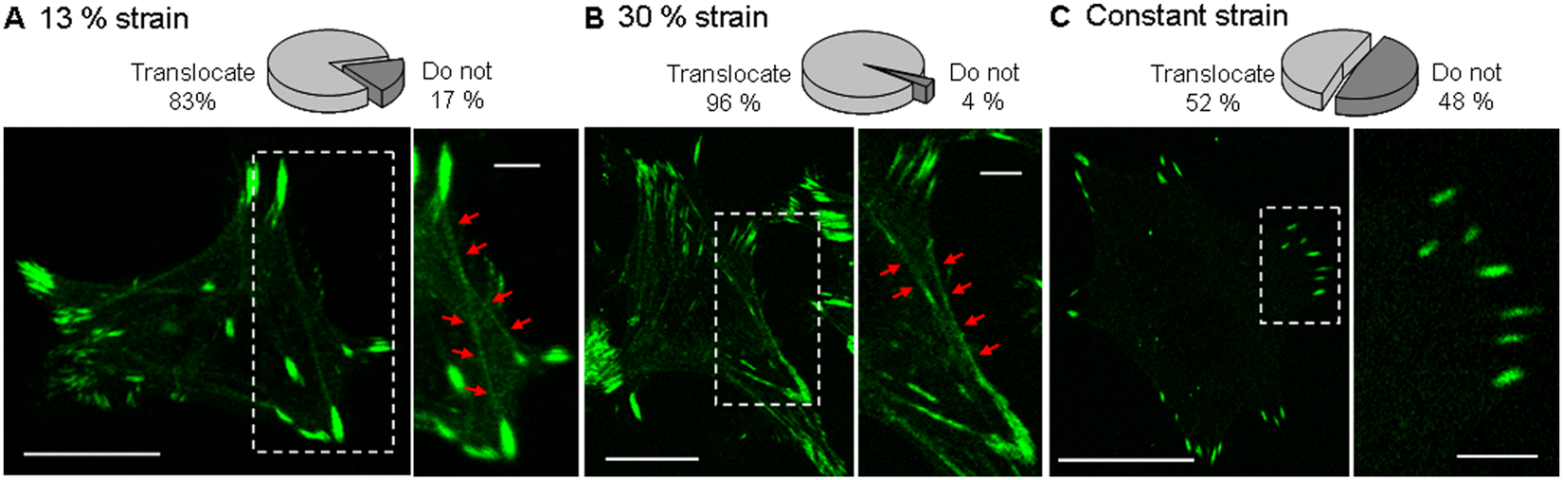
Zyxin translocation in response to mechanical strain. Representative confocal images of HC11 cells expressing zyxin-EGFP at 13% **(A)**, 30% normal strain **(B)**, and cultured on coverglass **(C)**. Besides its location at focal adhesions, in both cases under strain **(A**,**B)**, zyxin is typically found to be located along actin filaments (indicated by red arrows); while in cells maintained at a constant strain, such as cells cultured on coverglass, zyxin has approximately the same probability of being found or not along actin filaments. In the case presented in **(C)**, zyxin was only located at focal adhesions. On top of each panel, is displayed the percentage of cells showing zyxin translocation to actin filaments. Entire cell images scale bars: 20 μm; zoomed images scale bar: 5 μm. N = 23 cells were analyzed at 13% of strain, N = 23 cells at 30% of strain, and N = 52 cells cultured on coverglass.

In order to evaluate if substrate deformation produced by the stretching device can alter zyxin localization in epithelial mammary cells, HC11 cells were transfected with the zyxin-EGFP construct. Stretching experiments were performed and confocal images were acquired at 13% or 30% normal strain. As a control of a non-deformable substrate, confocal images of cells cultured on coverglass were also acquired. Zyxin in HC11 mammary cells was found to be distributed along stress fibers in addition to its conventional location at focal adhesions, in stretching experiments as well as in control experiments. Interestingly, zyxin translocation was observed in a basal population of approximately 50% in HC11 mammary cells cultured in a rigid substrate but without mechanical stimulation. However, after equibiaxial stretching, the recruitment of zyxin to stress fibers was significantly increased: translocation of zyxin-EGFP was observed in 83% and 96% of the analyzed stretched cells at normal strains of 13% and 30%, respectively (Fig. 5A,B), while only in 52% of the HC11 cells cultured on coverglass (Fig. 5C). These results suggest that the recruitment of zyxin to stress fibers not only depends on the substrate rigidity (which here is maximum on coverglass), but also is a dynamic response to an increase of global cell stretching, in accordance with its restoration function.

### Equibiaxial strain alters zyxin unbinding kinetics within focal adhesions

Molecular binding kinetics of zyxin, as well as that of a number of focal adhesion proteins, is also known to be altered by mechanical forces^40^. The dissociation constant (${k}_{off}$) of individual proteins from the adhesion complex is a key determinant of the turnover of focal adhesions and is proven to be sensitive to changes in applied forces^40^. In particular, the force-dependent changes in zyxin kinetics have been previously shown by modulating cytoskeletal tension in different ways. Dissipating contractile forces exerted by the actin cytoskeleton using ROCK inhibitors, resulted in an increase of the dissociation constant of zyxin from focal adhesions^41^. A similar effect was observed when traction force at adhesions was physically disrupted by laser ablation of individual stress fibers anchored into the adhesion^41^. The culture of cells on substrates of decreasing stiffness showed that zyxin in adhesions formed on soft substrates had higher ${k}_{off}$ values than those on stiff substrates^41^.

To study the molecular binding kinetics of zyxin to focal adhesions, we used an approach based on Fluorescence Recovery After Photobleaching (FRAP)^42,43^ on living HC11 cells expressing zyxin-EGFP construct. Employing the confocal laser spot, a small area within a focal adhesion was photobleached. The diffusion of unbounded molecules was assumed to be much faster than the binding kinetics, and the concentration of freely diffusing molecules was considered spatially homogeneous and temporally constant during the course of the FRAP experiment. Under these conditions, the fluorescence recovery is dominated by the reaction of dissociation of the protein to the focal adhesion, so the normalized recovery curves were fit by equation (8) and the unbinding constant ${k}_{off}$ and mobile fraction *m* were obtained^44^. This approach provides an ‘effective’ ${k}_{off}$ representative of the unbinding interactions of the proteins that may interact with multiple different partners in the focal adhesion complex.

**Figure 6:**
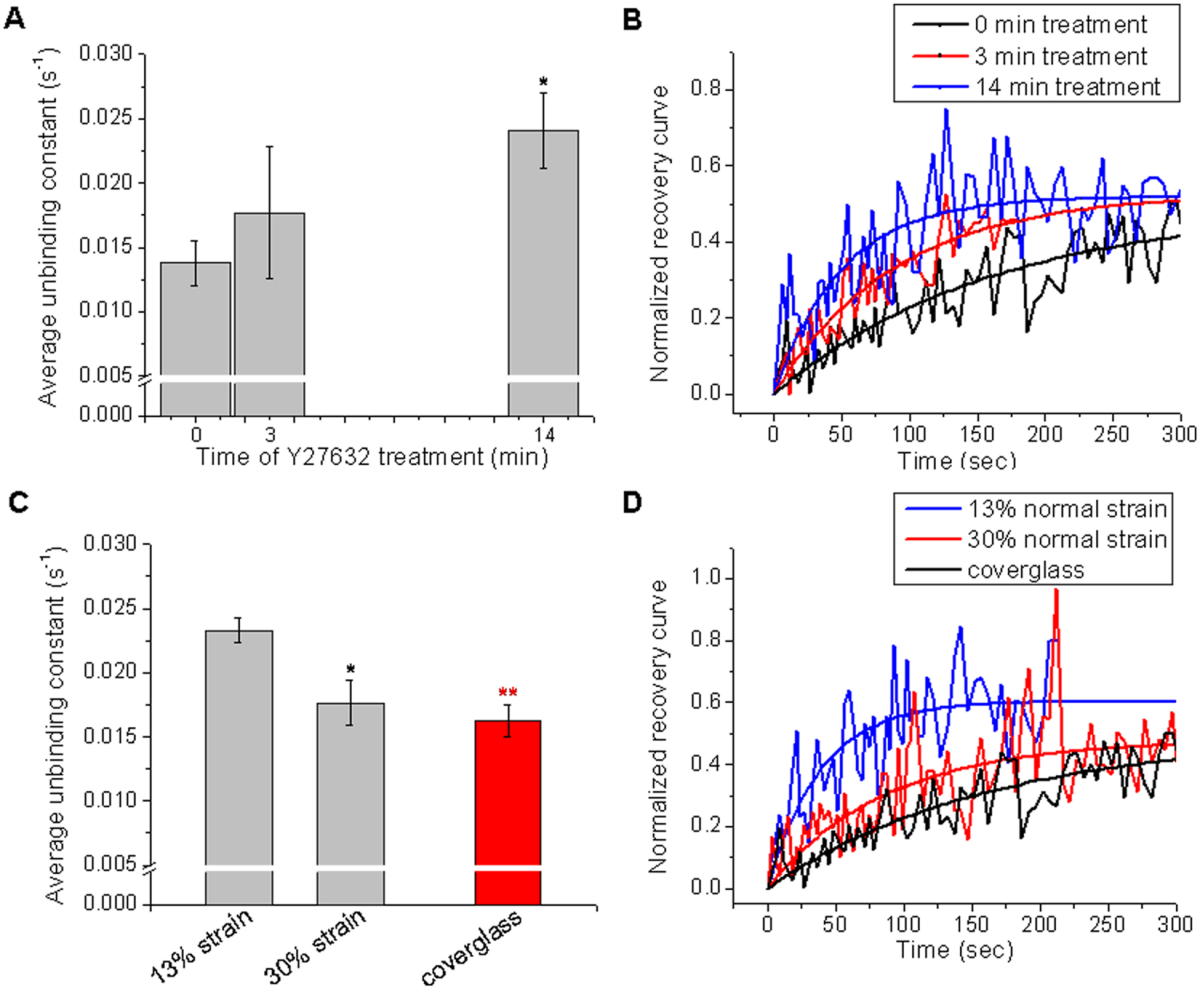
Zyxin unbinding kinetics in response to strain evaluated by FRAP. (**A**) Average values of the unbinding constant of zyxin as a function of the exposure time to Y-27632 (error bars indicate s.e.m.; N = 7, N = 3 and N = 4 recovery curves, for 0, 3 and 14 min after treatment; Mann-Whitney test, *p-value = 0.012 compare to t = 0 min). **(B)** Recovery curves for zyxin in control (black) and Y-27632-treated cells for 3 min (red) and 14 min (blue), together with the curves fit to the data using the FRAP model for focal adhesions described in Materials and Methods. **(C)** Average values of the unbinding constant of zyxin as a function of normal strain. As a rigid substrate control, FRAP experiments were performed in cells grown on coverglass. N = 6 recovery curves were analyzed from different focal adhesions of 5 and 6 cells at 13% and 30% strain, respectively. N = 12 curves from 8 cells cultured on coverglass; error bars indicate s.e.m.; Mann-Whitney test, *p-value = 0.026, **p-value = 0.00075, both compared to 13% strain. **(D)** Representative recovery curves for zyxin in cells under 13% normal strain (blue), 30% normal strain (red) and grown onto a rigid substrate (black), together with the curves fit to the data using the FRAP model for focal adhesions described in Materials and Methods.

The applicability of this approach to follow ${k}_{off}$ mechanoresponses in HC11 cells, was confirmed by experiments disrupting internal cell tension using the ROCK kinase inhibitor Y-27632, which dissipates cytoskeletal tension^45^. To this end, FRAP experiments were performed in HC11 cells cultured on coverglass and treated with Y-27632. Figure 6A shows the average unbinding constant of zyxin as a function of the exposure time to Y-27632. As the exposure time increased, cytoskeletal tension diminished and the unbinding constant of zyxin was found to increase. After 14 min of treatment, ${k}_{off}$ was significantly higher compared to the 0 min treatment, as can also be seen in the representative recovery curves from Fig. 6B. Decreasing the internal force exerted on the focal adhesions by the inhibitor treatment caused indeed a faster fluorescent recovery of zyxin molecule into the photobleached region.

To investigate the influence of equibiaxial stretching on the molecular binding kinetics of zyxin, we used the same approach in HC11 cells stretched at 13% and 30% strain and in cells cultured on coverglass. Figure 6C shows the average values of the unbinding constant of zyxin as a function of strain, as the stretching is increased from 13% to 30%, the dissociation constant of zyxin diminishes significantly. At 30% strain, the obtained ${k}_{off}$ is similar to the observed in cells grown in a rigid substrate as the fibronectin coated coverglass. Figure 6D shows representative normalized recovery curves obtained at the different mechanical conditions studied, which also evidence the time response of the fluorescence recovery of zyxin in the photobleached region. On the other hand, the average mobile fraction did not change significantly at the different studied conditions (data not shown).

At the opposite side of experiments relying on internal cell tension inhibition, our stretching experiments allow quantifying changes in zyxin kinetics in response to an increase in global cell tension. We found that zyxin dissociation from the focal adhesions is significantly faster for cells under low normal strain compared to cells under higher strain or grown on coverglass. In both cases, either disrupting internal tension with the Y-27632 inhibitor or increasing cellular tension by mechanically stretching, our findings suggest that higher cellular forces correlates with zyxin slower recruitment times, which is in concordance with our previous results on focal adhesion stabilization and growth under force.

## Discussion

Mechanical signals are proposed to have a direct role in the significant changes experienced by the mammary gland epithelium during the successive stages of pregnancy, lactation and involution^20,46,47^. In this work, we exerted sustained equibiaxial mechanical strain to living mammary cells using a cell-stretching device adapted for live cell fluorescence imaging. This stretching device showed a linear and homogeneous response in both normal strain directions and allows exerting up to 30% of normal strain and a maximum normal stress of 0.85 MPa, which is efficiently transmitted from the elastic substrate to the attached cells as confirmed by cell area changes.

In an effort to get light into the mechanical signaling involved in the mammary gland, we used this device to exert a controlled and uniform strain to HC11 epithelial mammary cells while evaluating dynamic, morphometric and molecular changes in focal adhesions. Interestingly, we found that equibiaxial cell stretching stabilize focal adhesions enhancing their persistency and preventing their disassembly. Stabilization of focal adhesions was previously found by inhibiting microtubules polymerization with nocodazole^48^, but was not directly related to equibiaxial mechanical strain at the time. However, our findings are in concordance with counterpart experiments which observed the focal contact disassembly in correlation with uniaxial stretching in a direction perpendicular to the long cell axis^11^, a retraction of the cell^22^, or a deficient cell spreading caused by low rigidity substrates^23^. Focal adhesions morphometric changes generated by an increasing equibiaxial mechanical strain were also evaluated. We observed that focal adhesion area and length increase in response to the equibiaxial stretching, and that this increase is over the mere increase due to the mechanical stretching of the substrate on which the cells are attached. The mechanically modulated growth of focal adhesions is in agreement with previous results obtained with different mechanical stimuli as shear stress^49^, uniaxial stretching^11^, locally applied forces^26,27,50^ or substrate rigidity^23^.

The use of a FRET-based vinculin tension sensor, allowed us to investigate changes generated in the tension across vinculin protein due to the stretching external stimulus. We quantitatively followed changes in vinculin molecular tension at focal adhesions in response to successive increments in equibiaxial strain, finding that tension across vinculin molecule tend to increase as normal strain increases. These results suggest that vinculin protein in focal adhesion senses the external stimulus applied by the stretching device by increasing their molecular tension. Several works have reported changes in tension across vinculin due to alterations in internal cellular forces, proving that reducing intracellular tension by either pharmacological inhibition or mechanical disruption, result in a decrease in vinculin tension. However, changes in tension across vinculin due to global mechanical external strain have to our knowledge not been reported before. Our findings on the response to externally applied physical stimuli, reinforce previous studies by correlating the global mechanical state of the cell with the molecular tension across the protein vinculin within focal adhesions. These results provide further support for the hypothesis that vinculin is one of the major force sensing and transmitting component of cell-matrix adhesion.

We explored as well the effect of an external equibiaxial strain on the localization and the dissociation kinetics of the adhesion protein zyxin, also postulated to be a mechanosensor. We found that substrate stretching favors zyxin translocation to stress fibers, a response commonly associated with the remodeling and restoration of stress fibers after mechanical stimulation either at a global cell level or by applying targeted forces. At the molecular level, we studied the dissociation constant (${k}_{off}$) of zyxin protein from the focal adhesion complex, which was already proven to be sensitive to changes in applied forces^40^. Changes in the dissociation constant of zyxin due to alterations of internal cellular forces have been reported on several works by reducing intracellular tension^41,42^. Zyxin ${k}_{off}$ was also found to have a correlation with the external mechanical context of the cell by studying cells cultured on substrates with different stiffness^41^, but in this type of approach the effect cannot be followed in the same cell. Here we combined for the first time, FRAP measurements and mechanical stretching to investigate changes in the dissociation constant of zyxin in response to strain. Using this approach we evaluated an effective ${k}_{off}$ for zyxin in cells subjected to increasing equibiaxial stretching, observing a significantly slower zyxin unbinding kinetics in cells stretched at 30% normal strain compared to cells subjected to 13% of strain. This slower dissociation of zyxin from the focal adhesions could be connected with the enhanced focal adhesion stability under force, which was here observed by an increased persistence of focal adhesions in cells under stress.

To conclude, using the stretching device, sustained equibiaxial mechanical stimuli were applied to living HC11 mammary epithelial cells, resembling physiological stimuli to which these cells are subjected in the live organism. In such a way, we have found that increasing normal strain, not only induce focal adhesions stability together with an increase in their size, but also increases tension across the vinculin molecule within focal adhesions and significantly decreases the dissociation rate of zyxin from the focal adhesion. Our findings provide new insights into focal adhesion dynamics in response to external mechanical stimuli, and may contribute to gain deeper understanding of how forces regulate molecular responses of cells under normal physiological mechanical stimuli such as in the mammary gland.

## Materials and Methods

### Equibiaxial Stretching device

To perform a mechanical stimulus we used a home made equibiaxial stretching device adapted from Quaglino *et al*.^20^ for live imaging in an Olympus FV1000 spectral confocal microscope (Olympus Co., Japan). The device applies controlled equibiaxial strain to cells attached to flexible membranes, by pulling a rigid plastic ring that forms the walls of the cell culture chamber, over the clamped membrane. More details on the principle of stretching based on indenter designs are described in^51–53^. The stretching device consists in an aluminum screw-top support that contains: a two cylindrical pieces membrane holder (Delrin® made) where the silicone membrane (40 mm diameter) is clamped; a Teflon® indenter ring (30 mm diameter) that forms the walls of the cell culture chamber; and a Delrin® made flange that pushes the indenter ring that stretches the silicone membrane when the aluminum screw-top is turned down. The degree of stretching can be characterized in terms of thread turns of the aluminum screw-top.

### Silicone membrane

Transparent silicone elastic membranes of 127 μm thick in its unstretched state (Gloss/Gloss non-reinforced silicone sheeting; Specialty Manufacturing Inc., Saginaw, MI, USA) were used as culture substrate, suitable for fluorescence microscopy. Considering the Shore A hardness of 40 durometer (ShA) informed by the manufacturer, the elastic membrane Young’ Modulus (E) was estimated in 1.54 MPa, from the relationship between hardness and stiffness^54^:

1 $$E=\frac{17.93\cdot ShA}{{(100-ShA)}^{3/2}}$$

### Mechanical strain calibration - Computation of Strain Field - PIV Analysis

Two-dimensional deformations were measured by tracking fluorescent microspheres (0.5 μm diameter Dragon Green, Ex 480 nm, Em 520 nm, Bangs Laboratories Inc, Fishers, IN, USA) attached to the silicone membrane at different stretching conditions. For this purpose, confocal images of the fluorescent microspheres attached to the membrane were taken at an increasing number of thread turns. From these images, the positions of the markers were tracked using the particle image velocimetry (PIV) algorithm employing the open-source MATLAB (MathWorks Inc., Natick, USA) code MatPIV V1.6.1 of Sveen, 2004^55^. This algorithm is based on cross-correlating image sub-regions between sequential pairs of images. By processing the images over a regular grid of small sub-regions, a displacement vector map is generated. To remove spurious vectors (in general due to image sub-regions with not enough or too many particles to create a good pattern for matching), a series of filters are applied: a signal-to-noise ratio filter, a global histogram filter, a local filter and a masking mode for neglecting regions without out fluorescent markers. Finally, all the identified outliers are interpolated using a nearest neighbor interpolation.

Once the displacement field was obtained, mechanical strains and stresses were calculated employing a custom-written MATLAB code. Normal strains ${\varepsilon }_{xx}$ and ${\varepsilon }_{yy}$ in $x$ and $y$ directions, and shear strain ${\varepsilon }_{xy}$ were calculated at every point in the grid assuming a linear elastic material^56^:

2 $$\begin{array}{c}{\varepsilon }_{xx}=\frac{\partial {u}_{x}}{\partial x}\\ {\varepsilon }_{yy}=\frac{\partial {u}_{y}}{\partial y}\\ {\varepsilon }_{xy}=\frac{1}{2}\cdot (\frac{\partial {u}_{x}}{\partial y}+\frac{\partial {u}_{y}}{\partial x})\end{array}$$

where the displacements in the $x$ and $y$ directions are denoted as (${u}_{x}$, ${u}_{y}$). Then, the mean and standard deviation of ${\varepsilon }_{xx}$, ${\varepsilon }_{yy}$ and ${\varepsilon }_{xy}$ were calculated and plotted against the corresponding number of thread turns. The relation between number of thread turns and strain was obtained from the linear regression fit of the mean normal strain as a function of thread turns. For an isotropic material obeying Hooke’s law, the strains ($\varepsilon$) are related to the stresses ($\sigma$) by^57^:

3 $$\begin{array}{c}{\sigma }_{xx}=\frac{E}{1-{\nu }^{2}}\cdot ({\varepsilon }_{xx}+\nu \cdot {\varepsilon }_{yy})\\ {\sigma }_{yy}=\frac{E}{1-{\nu }^{2}}\cdot (\nu \cdot {\varepsilon }_{xx}+{\varepsilon }_{yy})\\ {\sigma }_{xy}=\frac{E}{1-{\nu }^{2}}\cdot \frac{1-\nu }{2}\cdot {\varepsilon }_{xy}\end{array}$$

where ${\sigma }_{xx}$ and ${\sigma }_{yy}$ are the normal stress in $x$ and $y$ directions, ${\sigma }_{xy}$ the shear stress, $E$ is the Young’s Modulus ($E=1.54\,MPa$) and $\nu$ the Poisson’s Radius ($\nu =0.5$). The reported strain and stress were obtained averaging the results of three different membranes stretching experiments.

### Cell culture

The HC11 cell line, derived from pregnant BALB/c mouse mammary glands, were grown in RPMI 1640 medium (Invitrogen – Gibco, Thermo Fisher Scientific Inc, Waltham, MA USA) supplemented with antibiotic-antimycotic (Invitrogen - Gibco), 10% fetal calf serum and 5μg of insulin from bovine pancreas per ml (Sigma Aldrich, St. Louis, MO). Silicone membranes were incubated for 30 min in 5 μg/ml fibronectin solution. Cells were seeded onto the fibronectin-coated membrane at a density of approximately 10 × 10^5^ cells/membrane and allowed to grow for 24 h, and then were transiently transfected using Lipofectamine® 2000 Reagent (Invitrogen). The following expression plasmids were transfected into the HC11 cells in order to visualize focal adhesions via the molecular scaffold protein vinculin: vinculin-EGFP (courtesy of Dr. B. Geiger), vinculin tension biosensor (Vin TS) or vinculin tail less (Vin TL). These two last constructions are based on the FRET pair mTFP and Venus (courtesy of Dr. C. Grashoff). In the case of the focal adhesion zyxin, the construction zyxin-EGFP (courtesy of Dr. D. E. Ingber) was used.

To dissipate cytoskeletal tension, cells were treated with Y-27632 (Caliochem®, San Diego, CA, USA), a highly potent, cell-permeable and selective inhibitor of Rho-associated protein kinases. Y-27632 inhibits p160ROCK (K = 140 nM) and also ROCK-II with equal potency^58,59^. For FRAP experiments employing the inhibitor, cells were cultivated onto coverglass previously coated with fibronectin, and Y-27632 was added to the culture media at a concentration of 10 µM.

### Microscopy - Image Acquisition

Fluorescent experiments were performed between 24 and 48 hs after transfection. The silicone membrane with the attached cells was mounted on the stretching device at a certain intensity of sustained equibiaxial strain (given by the number of thread turns) and 2 ml of culture medium with HEPES was added. By increasing the number of thread turns the membrane was exposed to successively increasing strain up to 30%. A microscope incubator chamber was used to maintain 37 °C temperature during experiments.

Live-cell microscopy was performed in a spectral confocal scanning microscope FluoView1000 (Olympus Co., Japan), employing either a 63X C-Apochromat water immersion NA 1.2 Zeiss or a 40X UPLSAPO NA 0.95 Olympus. For PIV Analysis a 10X UPLSAPO NA 0.4 was used. In the case of cells cultivated on coverglass a 60X UPLSAPO oil immersion objective lens NA 1.35 was employed. Images were obtained using Kalman filtering, pixel size was between 88 nm and 165 nm, and the time per pixel between 10 μs to 20 μs. For monitoring EGFP the 488 nm line of the Argon laser was used and the emitted fluorescence was detected at [500–600] nm. When the Vin TS or Vin TL were employed, 458 nm line of the Argon laser was used and the emitted fluorescence of mTFP and Venus were detected in the [470–500] nm and [530–600] nm ranges, respectively.

### Focal adhesion detection and morphometric analysis

For the morphometric analysis, vinculin was used as a focal adhesion marker. When the Vin TS or Vin TL constructs were employed the average image of the two channels detected was used for further analysis. Confocal image sequences of cells expressing a tagged version of vinculin and cultivated in the silicone membranes were obtained at successive strain increments from 13% to 30%. As a reference, control image sequences were obtained as a function of time at a constant strain of 13%.

For studying focal adhesion morphometry we have developed a custom-written MATLAB code (MathWorks Inc., Natick, USA), that allows semi-automatic identification of focal adhesions and quantitative morphometric analysis. This code was applied to image sequences of both control and stretching experiments. For each image of a sequence the background level is characterized by determining the average intensity and standard deviation over a region of the image without cells. The average background level is then subtracted to the entire image, and the standard deviation is employed as a threshold to identify cell pixels from background pixels (cell threshold was between 0.75 to 2 times the background standard deviation). From each image, over all the pixels of the identified cell, the Otsu’s method is applied to automatically calculate an optimum threshold for focal adhesions segmentation. This algorithm assumes that the image contains two populations of pixels (in this case: focal adhesion pixels and the rest of the cell pixels) and then calculates the optimum threshold separating those two classes so that their combined spread (intra-class variance) is minimal^60^. A binary pre-focal adhesion mask for identifying focal adhesion pixels is calculated employing twice the Otsu threshold. This mask is segmented into 2D structures of connected white pixels. Structures with smaller areas than 20 pixels (approx. 0.25 μm^2^) and pixels with less than 3 white neighbors are discarded. Finally, focal adhesions are manually labeled and holes in any single adhesion were filled. After focal adhesion identification, characteristic adhesion properties, such as area, length, eccentricity and mean intensity are calculated for every image of a sequence. The focal adhesion mean intensity is divided by the mean fluorescence intensity of the entire cell to taking into account the acquisition photobleaching. Focal adhesion length and eccentricity are defined as the length of the major axis and the eccentricity of the ellipse that has the same normalized second central moments as the focal adhesion region.

A total number of 55 or 19 persistent focal adhesions from 4 cells were analyzed for stretching or control time lapse experiments, respectively.

### FRET experiments and analysis

FRET experiments were performed in HC11 cells expressing a vinculin tension sensor (VinTS) which incorporates a tension sensor module, inserted after amino acid 883 of vinculin. The tension sensor module consists of a FRET pair fluorophores, mTFP1 and Venus, separated by a flagelliform linker sequence (GPGGA)_8_. When force across this construct extends the elastic linker, FRET efficiency decreases. As a positive FRET control, vinculin tail less (VinTL), a similar construct without the vinculin tail domain, was employed. Simultaneous confocal images of the donor (mTFP1) and acceptor (Venus) were acquired at increasing normal strains from 13% up to 30%, employing a 458 nm line of the Argon laser. The emitted fluorescence of mTFP1 and Venus were detected in the [470–500] nm and [530–600] nm range, respectively. Changes in FRET efficiency were quantified through a ratiometric approach, particularly suited for intramolecular FRET sensors^61^. For each detected focal adhesion, a FRET ratio value ($R$) was calculated as the ratio between the mean fluorescence intensity of the acceptor (Venus, ${I}_{Venus}$) and the donor (mTFP1, ${I}_{mTFP}$):

4 $$R=\frac{{I}_{Venus}}{{I}_{mTFP}}$$

For each strain condition, a mean focal adhesion FRET ratio was calculated, averaging the FRET ratio of N = 53–80 focal adhesions from 6 cells expressing Vin TS and of N = 21 focal adhesions from 2 cells expressing the Vin TL construct.

The normalized mean focal adhesion FRET ratio was calculated according to:

5 $$Normalized\,FRET\,ratio=\frac{R-{R}_{\min }}{{R}_{\max }-{R}_{\min }}$$

where ${R}_{\max }$ is the maximum FRET ratio value (estimated from control experiments employing the Vin TL construct) and ${R}_{\min }$ is the minimum FRET ratio value due to the spectral crosstalk of the donor signal into the acceptor channel (estimated from control experiments using cells expressing a vinculin construct without the acceptor, Vin mTFP). ${R}_{\max }$ was calculated averaging the FRET ratio of N = 21 focal adhesions from 2 cells expressing Vin TL at different stretching conditions from 4% up to 30% normal strain: ${R}_{\max }=2.81\pm 0.06$. ${R}_{\min }$ was measured from N = 13 focal adhesions from 2 cells expressing Vin mTFP, and corresponds to the ratio due to the bleed-through of the donor signal into the acceptor channel: ${R}_{\min }=0.64\pm 0.02$. The values ${R}_{\max }$ and ${R}_{\min }$ are entirely dependent on the spectral and detection configuration of the microscope, and must be measured for each system.

### FRAP experiments and analysis

FRAP experiments were performed in cells expressing zyxin-EGFP maintained at a sustained strain of 13% or 30%. As control experiments, cells cultivated on fibronectin coated coverglass were employed. For FRAP experiments, a selected small region (0.2–0.5) μm^2^ in a focal adhesion is photobleached employing 100% laser transmission of the 488 nm Argon line (approximately 0.5 mW) during 40–200 ms. Before bleaching, 5 control images are taken and immediately after bleaching a sequence of images is captured every 2 seconds.

The mean fluorescence intensity within the selected bleached region is monitored as a function of time, ${I}_{raw}$. For each image, the mean fluorescence intensity of a region without cells, ${I}_{bkgd}$, is calculated and, to minimize the effect of photobleaching during acquisition, ${I}_{raw}$ was divided by the mean fluorescence intensity of a control region (of the same area and in a focal adhesion not photobleached), ${I}_{control}$, obtaining:

6 $${I}_{FRAP}=\frac{{I}_{raw}(t)-{I}_{bkgd}}{{I}_{control}(t)-{I}_{bkgd}}$$

Then the recovery curve is calculated as:

7 $$F(t)=\frac{{I}_{FRAP}(t)-{I}_{o}}{{I}_{PRE}-{I}_{o}}$$

where ${I}_{PRE}$ is the intensity in the bleached region before photobleaching, ${I}_{o}$ is the intensity in the bleached region immediately after photobleaching. Only recovery curves with more than 90% intensity decrease immediately after photobleaching were analyzed. Recovering curves showing more than 20% of intensity decay due to unwanted photobleaching during acquisition were discarded.

Assuming that diffusion of unbound zyxin in the cytoplasm is much faster than its binding/unbinding kinetic to the focal adhesion, and photobleaching of cytoplasmic protein is negligible, the recovery during FRAP is purely due to recovery in the bound concentration of protein to the focal adhesion^43,44,62^. FRAP experiments on cytoplasmic regions -where zyxin is not bound to the focal adhesion- corroborated that the contribution of the unbound protein diffusion is negligible to the FRAP recovery curve (data not shown), supporting that the recovery during FRAP experiments within a focal adhesion is mainly due to the recovery in the bound concentration of zyxin.

When the kinetics of dissociation is rate limiting, the unbinding constant of zyxin, ${k}_{off}$, is determined by fitting (least-squares best fit) FRAP recovery data to the expression^44^:

8 $$F(t)=m\cdot (1-{e}^{-{k}_{off}\cdot t})$$

where $m$ is the mobile fraction. FRAP experimental recovery curves from 6 focal adhesions of 5 and 6 different cells at 13% and 30% strain respectively, were analyzed. The average unbinding constant and the mobile fraction were calculated together with their standard deviation.

### Data availability

The datasets generated and analysed during the current study are available from the corresponding author on request.
